# Equol and Resveratrol Improve Bone Turnover Biomarkers in Postmenopausal Women: A Clinical Trial

**DOI:** 10.3390/ijms241512063

**Published:** 2023-07-27

**Authors:** Graziamaria Corbi, Vincenzo Nobile, Valeria Conti, Alessandro Cannavo, Vincenzo Sorrenti, Alessandro Medoro, Giovanni Scapagnini, Sergio Davinelli

**Affiliations:** 1 Department of Translational Medical Sciences, University of Naples “Federico II”, 80138 Naples, Italy; graziamaria.corbi@unina.it (G.C.); alessandro.cannavo@unina.it (A.C.); 2 Farcoderm S.r.l., 27028 San Martino Siccomario, Italy; vincenzo.nobile@complifegroup.com; 3 Department of Medicine Surgery and Dentistry, Scuola Medica Salernitana, University of Salerno, 84081 Baronissi, Italy; vconti@unisa.it; 4 Department of Pharmaceutical and Pharmacological Sciences, University of Padua, 35131 Padua, Italy; vincenzosorrenti88@gmail.com; 5 Department of Medicine and Health Sciences “V. Tiberio”, University of Molise, 86100 Campobasso, Italy; alessandro.medoro@unimol.it (A.M.); sergio.davinelli@unimol.it (S.D.)

**Keywords:** equol, resveratrol, menopause, bone metabolism markers, bone mineral density

## Abstract

Estrogen deficiency is a major cause of loss of postmenopausal bone mineral density (BMD). This study aimed to evaluate the effects of equol and resveratrol on bone turnover biomarkers in postmenopausal women. Sixty healthy postmenopausal women were randomly assigned to receive 200 mg fermented soy containing 10 mg equol and 25 mg resveratrol or a placebo for 12 months. Whole-body BMD and bone turnover biomarkers, such as deoxypyridinoline (DPD), tartrate-resistant acid phosphatase 5b (TRACP-5b), osteocalcin, and bone-specific alkaline phosphatase (BAP), were measured at baseline and after 12 months of treatment. At the end of treatment, DPD, osteocalcin, and BAP significantly improved in the active group (*p* < 0.0001 for all) compared to the placebo group. Conversely, TRACP-5b levels were unaffected by supplementation (*p* = 0.051). Statistically significant changes in the concentrations of DPD (*p* < 0.0001), osteocalcin (*p* = 0.0001), and BAP (*p* < 0.0001) compared to baseline were also identified. Overall, the intervention significantly increased BMD measured in the whole body (*p* = 0.0220) compared with the placebo. These data indicate that the combination of equol and resveratrol may positively modulate bone turnover biomarkers and BMD, representing a potential approach to prevent age-related bone loss in postmenopausal women.

## 1. Introduction

The prevalence of age-related bone loss is higher in women than in men, especially postmenopausal women. This loss is typically associated with osteoporosis, a common chronic disease responsible for reduced bone strength, disruption of bone architecture, and an increased risk of fracture. In postmenopausal women, the incidence of hospitalization due to osteoporotic fractures is higher than that of stroke, myocardial infarction, and breast cancer. After the age of 50 years, approximately 50% of women have at least one fracture due to osteoporosis [1]. The consequences of osteoporotic fractures include a poor quality of life, disability, and mortality. Therefore, the clinical and economic burden of osteoporosis is increasingly being recognized as a serious public health problem [2].

Accelerated loss of bone mineral density (BMD), which occurs during menopause due to estrogen deficiency, is a key contributor to the incidence of osteoporotic fractures [3]. Although it is well established that fracture risk is higher in menopausal women with low BMD, considerable attention has been paid to the role of bone turnover biomarkers in the clinical evaluation of osteoporosis. These biomarkers are released during bone remodeling processes and fall into two categories: markers of bone formation produced by osteoblastic cells or derived from procollagen metabolism and markers of bone resorption, which are degradation products of osteoclasts or collagen degradation [4,5]. Although several biochemical markers of bone formation are currently in use, the measurement of osteocalcin and bone alkaline phosphatase (BAP) is recognized as a useful means for the management of osteoporosis and for the prediction of fracture risk in postmenopausal women [6]. To evaluate the response to pharmacologic treatment and to assist the clinical diagnosis of osteoporosis, among the most common markers of bone resorption, tartrate-resistant acid phosphatase 5b (TRACP-5b) and deoxypyridinoline (DPD) have shown great potential [5,7].

Recently, significant advances have been made in the development of nutraceutical agents to prevent age-related bone loss. Phytoestrogens are a group of plant polyphenols that are commonly used for the treatment of menopause-related conditions. They can be classified into four classes: isoflavones, lignans, coumestans, and stilbenes. The estrogenic activity of these compounds may exert a multitude of benefits in estrogen-deficient menopausal women, including beneficial effects on osteoporotic bone loss [8]. The health benefits of isoflavones in middle-aged women appear to be due to individual differences in their ability to produce equol in the intestine. In Western countries, only 30–50% of individuals metabolize the isoflavone daidzein into equol and are known as equol producers [9]. Some studies have reported that supplementation with equol contributes to improving bone health in postmenopausal women, including those who are not equol producers [10,11]. It was also shown that a combination of equol and resveratrol might attenuate menopausal symptoms and increase mitochondrial function [12,13]. Resveratrol is a phytoestrogen that modulates estrogenic activity through various mechanisms. It directly interacts with the estrogen receptor, acting as both an agonist and antagonist. This compound also affects the metabolism of estrogens in the intestines and liver, increasing their levels and enhancing their central and peripheral actions. Additionally, resveratrol inhibits key enzymes involved in steroidogenesis, influencing the plasma levels of steroids and their precursors. Resveratrol also exerts potent bone-protective effects, especially in experimental models that mimic postmenopausal osteoporosis caused by estrogen deficiency [14,15].

This randomized, placebo-controlled trial evaluated the efficacy of the combined use of resveratrol and equol on bone turnover biomarkers and whole-body BMD in postmenopausal women.

## 2. Results

**Figure 1:**
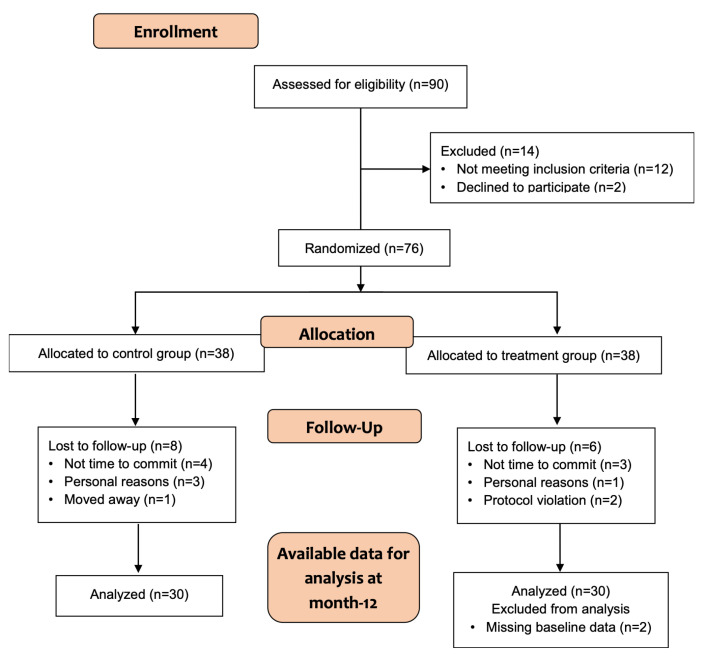
Flowchart of enrolment, intervention allocation, and data analysis.

The flowchart illustrating the study design is shown in Figure 1. A total of 76 healthy postmenopausal women were randomly allocated to receive the equol/resveratrol combination or placebo. At the end of the 12-month intervention phase, eight women in the placebo group and six in the active group were lost to follow-up. In the final analysis, two participants in the treatment group were excluded because the baseline data were lost and could not be recovered. The treatment was well tolerated by the patients, and none of the subjects experienced serious adverse events.

The characteristics of the participants are listed in Table 1. No significant baseline differences were observed between the active and placebo groups regarding the concentrations of osteocalcin, BAP, TRACP-5b, and DPD. All 60 participants who completed the study had a normal BMD at baseline. No baseline differences in whole-body BMD were found between the two groups. The control and intervention groups showed no change in compliance at baseline and follow-up.

**Figure 2:**
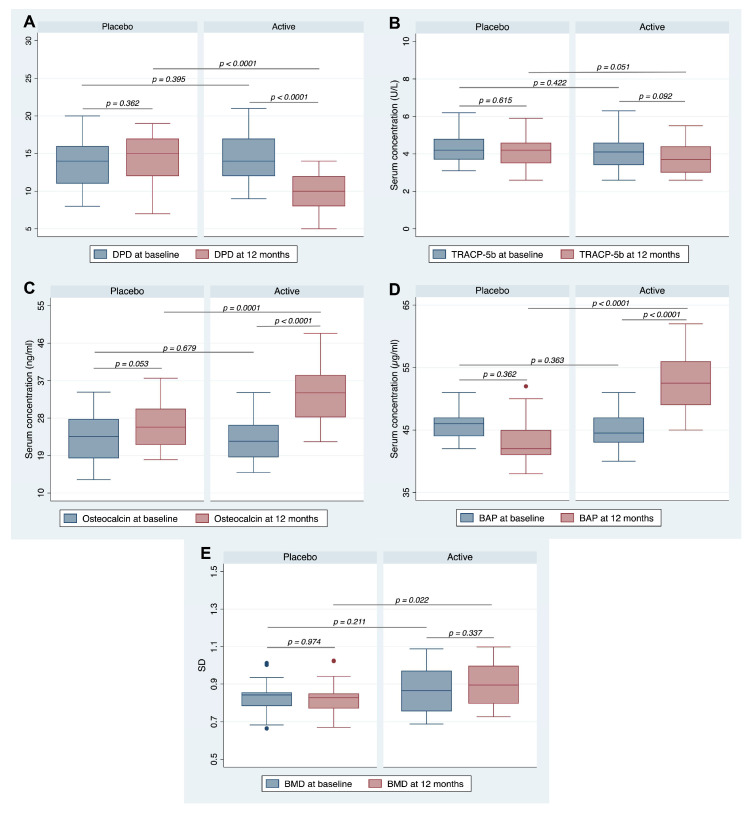
Concentrations of bone markers and BMD levels in the groups at baseline and after 12 months. The figure shows the concentration of (**A**) DPD, (**B**) TRACP-5b, (**C**) OC, (**D**) BAP levels, and (**E**) BMD measured in the whole body at baseline and after 12 months for both groups. The dots represent the outliner values.

After 12 months, the active group showed a statistically significant reduction in DPD levels from baseline (*p* < 0.0001, Figure 2A). Likewise, a significant change in the concentrations of OC and BAP compared to both the baseline and placebo (*p* ≤ 0.0001 for all, Figure 2C,D) was observed after 12 months. Conversely, TRACP-5b levels were unaffected by the supplementation. However, after 12 months, the active group showed a trend toward a reduction in TRACP-5b levels compared to the placebo group (*p* = 0.051, Figure 2B). The intervention significantly increased the BMD measured in the whole body (*p* = 0.0220, Figure 2E) compared to the placebo.

**Figure 3:**
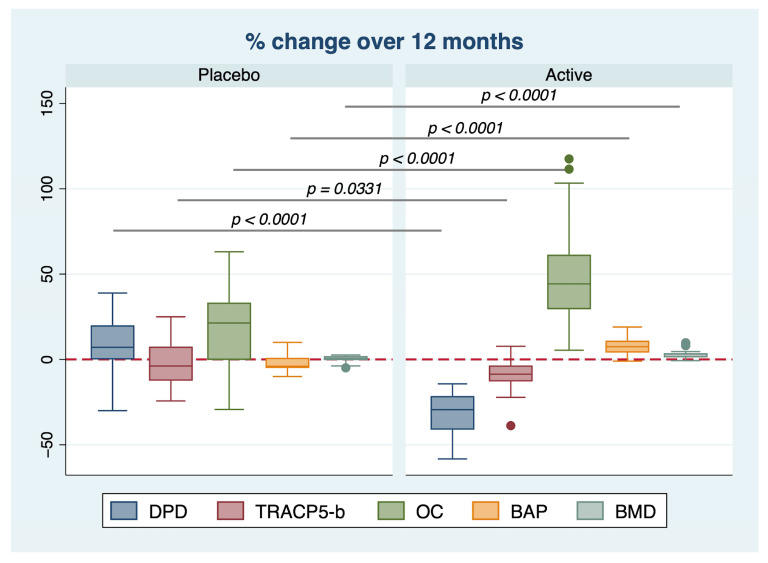
Percentage changes in all osteoblastic/osteoclastic parameters and BMD between the placebo and active groups were evaluated. The percentage changes in all bone metabolism markers and BMD were significantly different between the placebo and active groups. The dots represent the outliner values.

To assess the efficacy of treatment, we calculated the percentage difference between the initial and final values. The percentage changes in all bone metabolism markers and BMD were significantly different between the placebo and active groups: DPD −32.63 ± 12.08%, *p* < 0.0001; TRACP-5b −8.78 ± 9.24%, *p* = 0.0331; osteocalcin +49.70 ± 28.54%, *p* < 0.0001; BAP +8.00 ± 4.64%, *p* < 0.0001; BMD +3.17 ± 2.74%, *p* < 0.0001 (Figure 3).

To confirm these findings and determine the best predictive factor for percentage changes in BMD, multivariable linear regression analysis was conducted (Table 2). The analysis examined whether percentage changes in OC, BAP, DPD, TRACP-5b, and being in the active group were significant predictors of percentage changes in BMD.

The fitted regression model was 0.495 + 0.216 × (% change in BAP) − 0.014 × (% change in OC) + 0.008 × (% change in TRACP-5b) + 0.052 × (% change in DPD) + 3.394 × (active group). The overall regression was statistically significant (r^2^ = 0.4650, F (5.54), *p* < 0.0001). The best predictor of the percentage change in BMD was represented by the percentage changes in BAP (β = 0.216, 95% CI 0.0814–0.3498, *p* = 0.002), followed by participation in the active group (β = 3.394, 95% CI 1.245–5.544, *p* = 0.003), and percentage changes in DPD (β = 0.0516, 95% CI 0.008–0.095, *p* = 0.020). The analysis revealed that treatment with the equol/resveratrol combination was directly and significantly associated with higher percentage changes in BMD compared with placebo.

**Figure 4:**
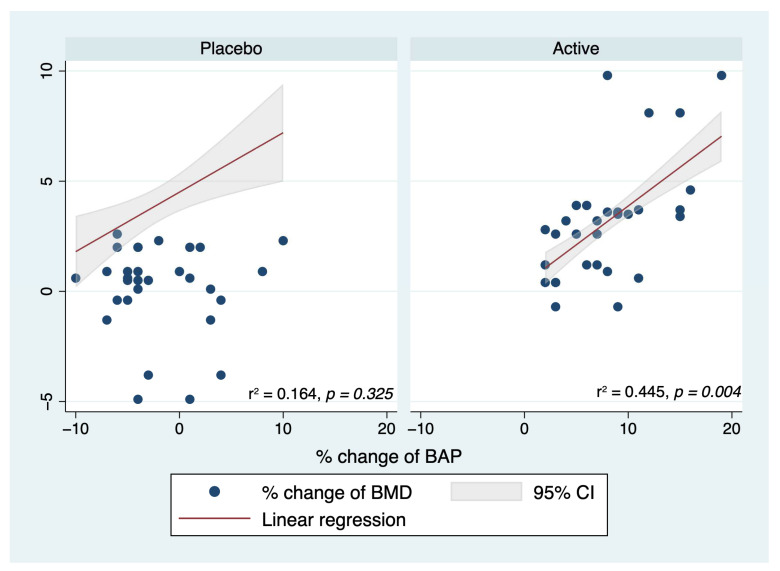
Linear regression analysis by group. In the placebo group, no predictors of changes in BMD were identified. Conversely, in the intervention group, a statistically significant association was found between the percentage changes in BAP and the percentage changes in BMD.

To better define the role of treatment in improving BMD, a linear regression analysis was performed by group. The analysis tested whether the percentage changes in OC, BAP, DPD, and TRPC-5b significantly predicted the percentage changes in BMD within each group. In the placebo group, no predictors of changes in BMD were identified. However, in the intervention group, a statistically significant association was observed between percentage changes in BAP and percentage changes in BMD (Figure 4) (β = 0.337, 95% CI 0.139–0.536, *p* = 0.002). This finding suggested that the equol/resveratrol combination acted favorably through the BAP to induce an increase in BMD.

## 3. Discussion

In this randomized placebo-controlled trial, supplementation with an equol/resveratrol combination for 12 months improved the bone anabolism in postmenopausal women. This supplementation promoted an increase in osteoblast markers (osteocalcin and BAP) and a decrease in osteoclast markers (DPD and TRACP-5b) (Figure 2 and Figure 3). These changes favorably counteracted the imbalance between excessive bone absorption and reduced bone formation in postmenopausal women. Moreover, these changes were also associated with an improvement in BMD, which generally exhibited a pathological reduction during menopause. These findings were confirmed by multilinear regression analysis, which identified the intervention group as a predictor of changes in BMD in a positive and direct manner (Table 2). When stratified by group, no association was found between changes in markers of bone absorption/resorption and BMD variations in the placebo group. In contrast, the active group showed a positive association between changes in BAP and BMD (Figure 4), suggesting that supplementation improved BMD by increasing BAP levels.

After menopause, women lose 3% of their BMD each year, resulting in osteopenia or osteoporosis [16]. Rapid BMD loss due to estrogen deficiency critically contributes to an increased incidence of fractures [3]. Although pharmacological options are available to counteract osteoporosis, considering the treatment duration for maintaining bone health during the postmenopausal period and the risks associated with the long-term use of estrogen therapy, it is clinically valuable to develop effective treatments with minimal side effects suitable for long-term use [17]. Therefore, dietary trends have been directed towards the utilization of health-protective and disease-preventive foods [18].

Equol is a daidzein metabolite produced by anaerobic bacteria with stronger estrogenic activity than any other isoflavone or isoflavone-derived metabolite [19]. Isoflavones have been repeatedly reported to help prevent osteoporosis [20,21], but the exact mechanism by which they preserve bone health is not completely understood. In a meta-analysis, Wei et al. found that soy isoflavones significantly increased bone mineral density by 54% and decreased the bone resorption marker urinary DPD by 23% compared to baseline in women. However, no significant effects on serum bone alkaline phosphatase activity have been observed [20]. In our study, the 12-month use of combined supplementation with equol and resveratrol was able to modify BMD and resorption and absorption bone markers.

Resveratrol has also been reported to act as a phytoestrogen. In a clinical study, resveratrol showed favorable effects on estrogen metabolism and circulating sex steroid hormones [22]. Recently, the Resveratrol for Healthy Aging in Women (RESHAW) trial demonstrated that a low dose of resveratrol (75 mg twice daily) supplementation in postmenopausal women, after 12 months, induced positive effects compared with placebo on bone density in the lumbar spine and neck of the femur. These effects were accompanied by a reduction in the levels of C-terminal telopeptide type-1 collagen, a bone resorption marker [23]. The significant benefits observed in our study may be the result of combining compounds, rather than the individual effects of equol or resveratrol. Although there are beneficial properties of equol and resveratrol against menopause-related symptoms, their effects on bone metabolism and prophylaxis of osteoporosis are poorly explored. To our knowledge, this is the first study to investigate the effects of combined supplementation of equol and resveratrol on bone metabolism in humans.

These compounds may act through multiple mechanisms, particularly through the estrogenic pathway. Resveratrol may exert its estrogen-like effect on bone by increasing the gene expression of osteoprotegerin, a protein that inhibits receptor activator of nuclear factor κB ligand (RANKL) to counteract osteoclast differentiation and activity [24]. Similarly, isoflavones have been reported to trigger the activity and proliferation of osteoblasts via insulin-like growth factor 1 (IGF-1), a key factor in maintaining bone mass against the action of osteoclasts [25]. In our study, multivariate analysis confirmed an association between the BMD improvement and increased BAP levels following supplementation, suggesting the involvement of this mechanism.

Soy isoflavones decrease RANKL levels and increase osteoprotegerin levels [26]. Therefore, isoflavones can improve bone metabolism and decrease bone resorption. Soy isoflavones decrease serum markers of bone resorption and improve bone metabolism. However, while the available data are promising, several studies have reported no change in RANKL and osteoprotegerin levels with isoflavone supplementation. In this regard, the current evidence is insufficient for conclusive approval of the efficacy of isoflavones in the RANKL/RANK/OPG pathway. Further research, including animal and human studies, is needed to confirm the effect of soy isoflavones on the RANKL/RANK/OPG pathway [26].

In most cases, supplementation included only isoflavones. Recently, Tousen et al. demonstrated that the combination of soy isoflavones and resveratrol prevented bone loss and decreased the RANKL/OPG gene expression ratio in bone marrow cells in unloaded mice [27], supporting the hypothesis of a beneficial multiplicative effect of these compounds on bone mass density.

Another factor that might have contributed to these effects could be related to the dosage. The dose of equol applied in this study has been used in several clinical trials and is reported to be effective for general menopausal symptoms without any serious adverse events. Tai et al. demonstrated that 24-month treatment with 300 mg/day isoflavones did not prevent the decline in BMD in postmenopausal women [28]. Kreijkamp-Kaspers et al. reported that a 12-month soy protein supplementation did not have any effect on BMD in postmenopausal women aged 60–75 years [29]. In contrast, Wu et al. assessed the positive effects of a 12-month intervention with soy isoflavones on the BMD of equol-producing postmenopausal Japanese women [30]. Consistent with our results, Wong et al. reported that a 12-month supplementation with 75 mg of resveratrol twice daily has the potential to slow bone loss in postmenopausal women [23].

The present study has several strengths and limitations. The main strength is rep-resented by the 100% retention rate for a 12-month intervention, associated with the investigation of the effects of simultaneous combined supplementation of equol and resveratrol on bone metabolism. The primary limitation of this study was its small sample size. However, several other similar studies did not include larger study populations. The second limitation is the duration of the study. Even though we conducted a 12-month study, a longer intervention duration may be helpful in further assessing the effects of equol and resveratrol on bone turnover and density. Finally, our study did not determine whether the enrolled subjects were equol producers. Therefore, even though the majority of Western women are not equol producers, we did not stratify the treatment and placebo groups based on equol-producing status.

In conclusion, our results suggest that combined supplementation of equol and resveratrol has some additional benefits on bone turnover biomarkers, especially in improving bone density formation in postmenopausal women. In the near future, larger studies should be conducted to confirm these results and investigate the molecular mechanisms underlying these effects. This non-pharmacological treatment represents a potential approach for preventing age-related bone loss in postmenopausal women.

## 4. Materials and Methods

### 4.1. Participants

Eligible subjects were all adult Caucasian menopausal women aged between 50 and 55 years old. The inclusion criteria were as follows: (1) menopause according to the WHO definition (after 12 consecutive months without menstruation) [31]; (2) case history characterized by menopausal complaints such as hot flushes, anxiety, emotional instability, sleep disorders, and depression; (3) 20 kg/m^2^ ≥ BMI < 25 kg/m^2^. Women were ineligible if any of the following criteria were present: (1) case history related to endometrial hyperplasia; (2) hormone replacement therapy (HRT); (3) metabolic syndrome; (4) pharmacological or nutritional treatments known to interfere with resveratrol and equol or affecting menopause symptoms. The subjects maintained their usual physical activity and diet pattern throughout the study. The study took place at Farcoderm S.r.l. facilities in San Martino Siccomario (PV), Italy. Subjects attended clinic visits at the time of randomization (baseline) and 12 months after supplementation initiation.

### 4.2. Study Design

A randomized, double-blind, placebo-controlled dietary intervention trial of 12 months duration was designed to evaluate the effects of equol and resveratrol supplementation on bone markers of in menopausal women. Of 90 eligible women, 12 were excluded because they did not meet the inclusion criteria, and 2 declined to participate (Figure 1). Thus, 76 menopausal women were included in the study. All the study procedures were carried out in accordance with the Declaration of Helsinki.

The study protocol was approved by the local ethics committee (study code: SI.02.DS.L; ref. no. 2011/3) and registered at the ISRCTN registry (ISRCTN10128742). All subjects provided written informed consent prior to the initiation of any study-related procedures.

### 4.3. Intervention and Randomization

The product was a commercially available dietary supplement (Equopausa D. Ulrich, Paladin Pharma S.p.A., Turin, Italy) containing 200 mg of fermented soy (including 80 mg of isoflavone aglycones and 10 mg of equol) and 25 mg of resveratrol from Vitis vinifera. The proposed level of equol intake has been used in several clinical trials and reported to be effective for menopausal symptoms [10,32,33]. The dosage of resveratrol was chosen because it was supported by several human studies showing that 25 mg of resveratrol per os was well absorbed and well tolerated [34,35,36]. Both active and placebo products were in tablet form and identical in appearance. They were prepacked in blisters and consecutively numbered for each subject according to the randomization list. Subjects’ compliance to treatment was assessed by means of product accountability as follows: at each visit, the expected number of consumed capsules was compared with the amount dispensed minus the quantity of the product that the subject returned.

Subjects were randomly assigned to receive the product or the placebo once a day. A restricted randomization list was created using PASS v.2008 (PASS, LLC. Kaysville, UT, USA) statistical software. The randomization sequence was stratified using a 10% maximum allowable % deviation with a 1:1 allocation ratio. The allocation sequence was concealed from the in-site study director in sequentially numbered, opaque, and sealed envelopes, reporting the unblinded treatment allocation. An independent technician dispensed either active or placebo products. Subjects, investigators, and collaborators were kept masked to product assignment.

The intervention period with phytoestrogens that would elicit a beneficial response in menopausal women is controversial and usually ranges from 3 to 12 months. Given that a short-term treatment of 3 months could have been a potential limitation, we chose 12 months of intervention as a in previous study investigating the effects of equol on bone metabolism in postmenopausal women [10].

### 4.4. Measurement of Bone Markers

Subjects were evaluated at baseline and after 12 months of treatment. Blood samples were centrifuged at 1200 g for 15 min at 4 °C, and serum samples were separated, divided into aliquots, and stored at −80 °C until analysis. Parameters of bone metabolism, such as OC and BAP, were measured by an electrochemiluminescence assay (ECLIA) (Roche Diagnostics, Mannheim, Germany). The serum level of TRACP-5b was measured using a fragment-absorbed immunocapture enzymatic assay (Quidel Corporation, San Diego, CA, USA). A complete 24 h urine collection was conducted at the beginning and end of the intervention and aliquots were stored at −80 °C until analysis. Urinary DPD concentrations were measured by high-performance liquid chromatography (HPLC) (Shimadzu, Kyoto, Japan).

### 4.5. Measurement of BMD 

The whole-body BMD was assessed by dual-energy X-ray absorptiometry (DXA) using a Lunar Prodigy Primo device (GE Healthcare, Chicago, IL, USA) with the Encore v.1 Windows XP software platform. The measurements were performed at baseline and after 12 months of treatment. The device was regularly calibrated before each diagnostic block and all DXA scans were clinically performed in accordance with the manufacturer’s recommendations.

### 4.6. Statistical Analysis

Categorical variables were summarized using frequencies and percentages, whereas, for continuous variables, means and standard deviations (SD) were used. For the univariate analysis, χ^2^ tests to compare categorical variables between the groups and the Student’s *t*-test for independent samples to compare continuous variables between the groups were used. To check the percentual change in all parameters, the following formula was used: [(Final value − baseline value)/baseline value] × 100. Multivariable linear regression was used to determine the independent predictive factors of % changes in BMD. Two-tailed *p* < 0.05 was considered statistically significant. Statistical analyses were performed using STATA 16 software (Stata Corp., College Station, TX, USA).

## Figures and Tables

**Table 1:** Baseline characteristics of study participants.

Characteristic	Placebo	Active	*p*
Age, years	52.69 ± 2.10	52.09 ± 1.71	0.931
SBP, mmHg	134.17 ± 12.87	134.26 ± 8.89	0.972
DBP, mmHg	85.17 ± 9.69	88.45 ± 8.46	0.171
BMI, Kg/m^2^	23.84 ± 2.19	23.42 ± 1.81	0.355
Total Cholesterol, mg/dL	171.38 ± 29.23	178.03 ± 30.33	0.322
HDL Cholesterol, mg/dL	49.60 ± 21.94	50.64 ± 20.71	0.818
LDL Cholesterol, mg/dL	91.24 ± 34.78	98.08 ± 34.31	0.379
Triglycerides, mg/dL	108.43 ± 62.97	110.11 ± 66.97	0.904
ALT, U/L	22.95 ± 5.93	24.13 ± 9.49	0.217
AST, U/L	23.57 ± 4.41	24.47 ± 5.25	0.406
Glycemia, mg/dL	93.60 ± 28.98	91.08 ± 16.74	0.635
DPD, pmol/μmol	13.73 ± 2.98	14.40 ± 3.05	0.395
TRACP-5b, U/L	4.29 ± 0.81	4.11 ± 0.91	0.422
Osteocalcin, ng/mL	23.53 ± 5.67	22.95 ± 5.20	0.679
BAP, μ/mL	45.77 ± 2.30	45.13 ± 3.00	0.363
BMD, SD	0.827 ± 0.097	0.867 ± 0.119	0.211

Abbreviations: SBP, Systolic blood pressure; DBP, Diastolic blood pressure; BMI, body. mass index; LDL, low-density lipoprotein; HDL, high-density lipoprotein; ALT, alanine aminotransferase; AST, aspartate aminotransferase; DPD, deoxypyridinoline; TRACP-5b, tartrate-resistant acid phosphatase 5b; BAP, bone-specific alkaline phosphatase; BMD, bone mineral density.

**Table 2:** Multivariable linear regression analysis.

Var_BMD	Beta	[95% Conf. Interval]		*p*
		Lowest	Highest	
Var_BAP	0.216	0.08	0.350	**0.002**
Var_OC	−0.014	−0.040	0.012	0.285
Var_TRACP-5b	0.008	−0.046	0.062	0.771
Var_DPD	0.052	0.008	0.095	**0.020**
Group				
Active	3.394	1.245	5.544	**0.003**

In bold the significant values are reported.

## Data Availability

The data presented in this study are available on request from the corresponding author. The data are not publicly available due to privacy.
